# Incidence de morsures de serpent dans les communautés rurales de savane de Paoua et de forêt de Mbaïki en République Centrafricaine

**DOI:** 10.48327/mtsi.v2i4.2022.211

**Published:** 2022-10-27

**Authors:** Romaric ZARAMBAUD, Germain PIAMALE, Jean de Dieu LONGO, Henri Saint-Calvaire DIEMER, Gérard GRESENGUET

**Affiliations:** 1 École doctorale des sciences de la santé humaine et vétérinaire, Université de Bangui, avenue des Martyrs, Bangui, République centrafricaine; 2 Département de santé publique, Faculté des sciences de la santé, Université de Bangui, avenue des Martyrs, Bangui, République centrafricaine

**Keywords:** Incidence, Morsures de serpent, Maladie tropicale négligée, Village, District sanitaire, Communauté rurale, Paoua, Savane, Mbaïki, Forêt, Lobaye, République centrafricaine, Afrique subsaharienne, Incidence, Snakebites, Neglected tropical diseases, Village, Sanitary district, Rural communities, Paoua, Savannah, Mbaïki, Forest, Lobaye, Central African Republic, Sub-Saharan Africa

## Abstract

**Introduction:**

L'objectif de cette étude est de décrire et de comparer les aspects épidémiologiques des morsures de serpent parmi les populations vivant dans le milieu rural de la zone de savane de Paoua et de la zone de forêt de Mbaïki en République centrafricaine (RCA).

**Matériels et méthodes:**

Il s'agissait d'une étude prospective à base communautaire dans deux districts sanitaires en RCA, de décembre 2019 à janvier 2021. Tout cas de morsure de serpent rapporté par les enquêteurs communautaires a fait l'objet d'un questionnaire afin de déterminer l'incidence, les circonstances de survenue, la gravité de la morsure et l'itinéraire thérapeutique.

**Résultats:**

Au total, 412 cas de morsures de serpent ont été recensés au cours de l’étude dont 198 cas dans la zone forestière et 214 dans la zone de savane. La létalité était de 5% dans la zone de forêt et de 1% dans la zone de savane.

Le taux d'incidence des morsures de serpent était nettement plus élevé chez les enfants de savane en comparaison avec ceux de la forêt (98/100 000 vs 25,1/100 000; p < 0,00001), tandis que ce taux d'incidence était nettement plus bas à partir de 45 ans dans la zone de savane de Paoua comparativement à la zone de forêt de Mbaïki (167/100 000 vs 200/100 000; p = 0,02). La létalité des enfants et des adultes jusqu’à 44 ans apparaissait nettement plus élevée en zone de forêt (7 décès vs 1 décès).

Ces morsures survenaient beaucoup plus lors des activités agricoles dans la zone de savane que dans la zone de forêt (51% vs 26%, p < 0,0001).

La symptomatologie était dominée par l’œdème et le saignement au point de morsure dans les deux zones d’études, compatibles avec un syndrome vipérin.

**Conclusion:**

Avec un taux d'incidence supérieur à 160 cas pour 100 000 habitants dans la population active de 15-44 ans des communautés rurales de la zone de forêt et de savane, les morsures de serpent restent un problème de santé publique en RCA. Une étude sur les espèces et la toxicité de leurs venins en RCA est recommandée. Par ailleurs, il est urgent de rendre disponibles les sérums antivenimeux dans les formations sanitaires périphériques afin de réduire la mortalité liée à ces envenimations.

## Abstract (en)

**Introduction:**

Snakebite is one of the most deadly neglected tropical diseases in Africa with more than 20,000 deaths reported each year. According to recent data from hospital in Central African Republic studies, the Paoua health district reports between 300 and 400 cases of ophidian envenomation each year. However, no epidemiological study on snakebites has been conducted at national level, nor a control strategy developed. The objective of this study is to describe the epidemiological aspects of snakebites in two rural communities, one located in savannah zone and the other in forest zone (both secondary and primary forest) in order to ensure adequate management of snakebites in these regions.

**Method:**

Prospective community-based study in two health districts in the Central African Republic, the health district of Paoua in savannah area and the health district of Mbaïki in forest area, from December 2019 to January 2021. Snakebites were investigated in the community by selected trained people in charge of reporting data regarding all known cases of snakebites occurring during the study period. The data were actively notified either by health personal or community health workers in order to determine the circumstances and severity of the bite, its management and the clinical course in case of envenomation.

**Results:**

A total of 412 snakebite cases were recorded during the study period, of which 198 cases occurred in the rural community of the forest zone and 214 in the community of the savannah zone. Case fatality rate was 5% in the forest zone and 1% in the savanna zone. The incidence rate of snakebite was significantly higher in savannah children compared to those in the forest (98/100,000 vs. 25.1/100,000; p<0.00001) while this incidence rate was significantly lower from age 45 onwards in the savannah area compared to the forest area (167/100,000 vs. 200/100,000; p=0.02). The case fatality rate of children and adults up to 44 years of age appeared to be significantly higher in the forest zone (7 deaths vs 1 death). Snakebites occurred significantly more frequently during field activities in the savannah zone than in the forest zone (51% vs 26%; p<0.0001). The symptomatology of bites was dominated by edema of the bitten limb and bleeding in the two study areas, compatible with cytotoxic and hemorrhagic syndromes due to viper bites.

**Conclusion:**

With an incidence rate of more than 160 cases per 100,000 inhabitants in the active population aged 15-44 years in rural communities of the forest and savannah zone, snakebites remain a public health problem in Central African Republic. A study on the toxicity of snakebites in Central African Republic is recommended. Besides, it is urgent to make anti-venomous serums available in health facilities in order to reduce the mortality related to the envenomation.

## Introduction

En Afrique intertropicale, les envenimations ophidiennes constituent une urgence médico-chirurgicale fréquente, quoique fortement sous-évaluée [9]. Même en l'absence de statistiques précises, il y aurait en Afrique plus d'un million de morsures de serpent par an, provoquant 600 000 envenimations aboutissant à plus de 20 000 décès [2]. Les trois-quarts des morsures surviennent au cours de travaux agricoles, de la chasse ou de déplacements pédestres en rapport avec le travail et la vie quotidienne [6, 8].

Du fait de sa position géographique à cheval sur deux zones climatiques, soudano-sahélienne au nord et équatoriale au sud, la République centrafricaine (RCA) bénéficie de conditions propices aux activités agropastorales.

La population ophidienne en RCA est composée de plus de 65 espèces de serpents dont certains sont venimeux comme *Bitis, Causus, Echis* de la famille des Viperidae, *Naja, Dendroaspis* de la famille des Elapidae, et *Atractaspis* de la famille des Lamprophiidae [12, 13].

Cependant, aucune étude épidémiologique n'a été réalisée afin de déterminer l'incidence et la létalité des envenimations de ces serpents. Toutefois, une équipe de Médecins Sans Frontières (MSF) déclare traiter 300 à 400 victimes de morsures de serpent par an à l'hôpital de Paoua [19].

Une meilleure connaissance de la morbidité et de la mortalité par morsures de serpent permettrait d'améliorer la prise en charge, l'accessibilité aux antivenins et de réduire la létalité et les séquelles [31].

L'objectif de ce travail est de décrire le profil épidémiologique et d'identifier les facteurs de risque de morsure de serpent en zone forestière dans le district sanitaire (DS) de Mbaïki et en zone de savane arborée dans le DS de Paoua afin d'améliorer les stratégies de lutte contre les envenimations ophidiennes.

## Materiels et Methodes

### Cadre de l’étude

L’étude a été réalisée dans les zones rurales de deux DS.

Le système de santé centrafricain se présente comme une structure pyramidale à trois niveaux dont la base représente le niveau le plus périphérique et le sommet le niveau central. En RCA, le district sanitaire comprend une population d'au moins 100 000 habitants, composée de communautés homogènes du point de vue socio-culturel. Il est constitué de l'hôpital de district et du réseau des Centres de santé et Postes de santé. La zone couverte par le district doit idéalement posséder une largeur maximale de 125 kilomètres. Le district sanitaire est l'unité opérationnelle du système national de santé, en dessous de la région sanitaire qui est le niveau intermédiaire et du niveau central selon la pyramide sanitaire [22, 28].

**Figure 1:**
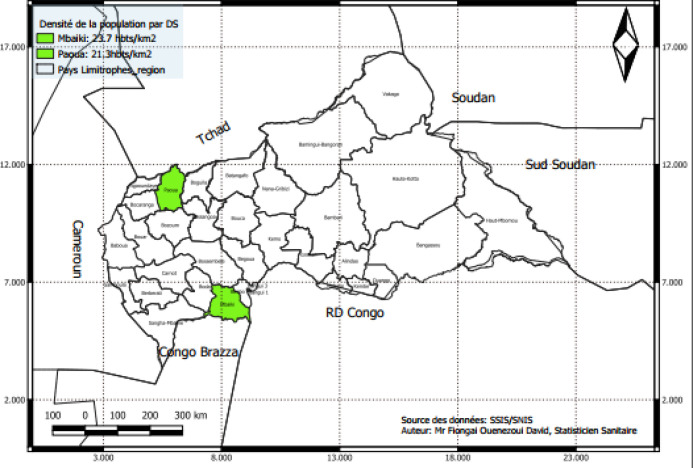
Cartographie des zones d’études (Source: SNIS) Mapping of the study areas (Source: SNIS)
- Le DS de Mbaïki situé dans le sud-ouest du pays est frontalier de la République du Congo. Il est situé dans la zone guinéenne forestière caractérisée par des précipitations moyennes annuelles supérieures à 1 600 mm. Les températures maximales moyennes annuelles varient entre 30 et 31 °C. La végétation est formée d'un côté de forêt dense semi-décidue à arbres géants caractéristiques de l'ancienne forêt primaire comprenant les communes de Nola, Balé-Loko et Mongoumba. De l'autre côté de savanes incluses situées au cœur de la forêt sur grés comme dans la commune de Balé-Loko au sud de la piste du 4^e^ parallèle (3°47’N-16°44’E) avec les villages de la SCAD (Société centrafricaine de déroulage) où se fait l'exploitation industrielle des grands arbres réputés abriter de nombreuses espèces de serpents. Les communes de Lesse et de Pissa avec les villages de Bossongo et de Centrapalm comptent 1853 ha en palmier à huile, 3649 ha en banane, 1687 ha en ananas et 71 ha en paddy qui sont aussi des gîtes de serpents [4, 12]. Deux types de cultures sont pratiquées: la culture vivrière et la culture industrielle. Il s'agit du manioc, de l'igname, du maïs, de l'arachide, du palmier à huile et du café. Les activités de cueillette sont une activité non négligeable et concernent les collectes de chenilles, de termites, de feuilles de koko *Gnetum africanum,* de champignons et de petits animaux nichés dans les terriers ou les trous d'arbres. Le calendrier saisonnier de ce district correspond à la saison sèche d'octobre à février où se fait la récolte du café et du manioc. La saison de pluie s’étend de mars à septembre, période de préparation du sol, de semis, de sarclage et de récoltes du maïs, de l'arachide et du riz ainsi que de cueillette des produits forestiers non ligneux.

- Le district sanitaire (DS) de Paoua est situé dans la partie nord-ouest de la République centrafricaine, frontalière au nord du Cameroun et au sud de la République du Tchad. Cette zone de savane est réputée abriter une forte prédominance de vipères *Echis romani* [12, 33]. Le climat est de type médio-soudanien avec une saison des pluies qui dure de mai à septembre, une pluviométrie oscillant entre 900 et 1 500 mm par an et une température moyenne de 28 °C [12, 15, 29]. La production agricole comprend du coton et des produits vivriers tels que manioc, arachide, maïs, riz, etc. La saison sèche est la période de récolte du maïs, du sorgho, du mil, du manioc et du coton ainsi que du ramassage des noix de karité. La saison pluvieuse est celle de la préparation du sol, des semis et du sarclage pour le coton et les cultures céréalières (Fig. 1).

### Méthode

Nous avons mené une étude prospective portant sur tous les cas connus de morsures de serpent durant la période de l’étude. Cette notification s'est faite dans le cadre d'une surveillance communautaire des cas de morsures de serpent par des enquêteurs formés. Cette surveillance consistait en le signalement et la notification de tout cas survenu dans la communauté par les enquêteurs communautaires et originaires de la région.

L'enquête a couvert la période allant de décembre 2019 à juillet 2020 dans les 7 communes du DS de Paoua (Bah-Bessar, Banh, Bimbi, Malé, Mia-Pendé, Mom et Nana-Barya), soit 8 mois, et de juillet 2020 à janvier 2021 dans les 8 communes du DS de Mbaïki (Mbata, Pissa, Bongo-Gaza, Lesse, Nola, Moboma, Balé-Loko), soit 7 mois.

La population de l’étude est constituée des enfants et adultes des deux sexes résidant d'une part dans les 7 communes des zones rurales et les 347 villages du DS de Paoua (population estimée en 2020 à 177 362 habitants, densité de 21 habitants/km^2^) et d'autre part dans les 8 communes des zones rurales et les 271 villages du DS de Mbaïki (population estimée en 2020 à 161 611 habitants, densité de 22 habitants/km^2^) [23]. Toutes ces populations sont considérées comme exposées au risque de morsure de serpent du fait qu'elles se trouvent dans des zones rurales.

Ont été incluses toutes les personnes des deux sexes, quel que soit leur âge, vivantes ou décédées, victimes de morsures de serpents pendant la période de l'enquête.

Il s'agissait d'un sondage exhaustif. Tout cas connu de morsure de serpent répondant aux critères d'inclusion a été admis dans l’étude.

### Collecte des données

Des séances de sensibilisation ont été préalablement réalisées à l'endroit des leaders communautaires (maires, chefs de groupes, chefs de villages, responsables religieux, etc.) sur l'objectif de l’étude et le signalement aux enquêteurs de tout cas de morsure de serpent survenu pendant la période de l'enquête. Des communications radiodiffusées dans les stations de radio communautaires sont réalisées une fois par semaine sur l'importance de signalement des cas de morsures de serpent aux enquêteurs communautaires, sur l'importance des soins médicalisés avec disponibilité des sérums antivenimeux gratuits au niveau des hôpitaux de district de Paoua et de Mbaïki. Ces émissions radiodiffusées étaient payantes au tarif de 15 000 FCFA, soit 30 dollars par mois.

Sept enquêteurs communautaires dans le DS de Paoua et huit enquêteurs communautaires dans le DS de Mbaïki, tous locuteurs de sango et de langues locales, originaires et habitants dans ces communes respectives, ont été recrutés localement et formés durant trois jours sur la documentation des cas de morsure de serpent auprès des victimes ou des parents de ces victimes, si la victime est un enfant.

Une pré-enquête pour tester l'assimilation des notions sur la morsure de serpent et le remplissage du questionnaire par ces enquêteurs communautaires avait été réalisée avant le démarrage de l'enquête proprement dite.

Ces enquêteurs communautaires étaient répartis à raison d'un enquêteur par commune. Ils collectaient les données à temps plein durant toute la période de l'enquête. Ils sillonnaient les villages à l'aide de vélos pour s'enquérir des cas de morsures de serpent afin de les notifier. Aussi, des cas de morsures de serpent leur ont été signalés par les leaders communautaires de bouche-à-oreille, soit par appel téléphonique, soit par messages radiophoniques afin qu'ils s'y rendent pour la documentation. Le délai moyen entre la morsure et la notification de cas par les enquêteurs était en moyenne de 24 heures.

Les chefs des différents centres de santé et les infirmiers diplômés d’État, membres des équipes cadres des districts sanitaires de Paoua et Mbaïki, étaient impliqués dans l'enquête comme superviseurs de ces enquêteurs communautaires afin de vérifier la fiabilité des informations fournies et de valider les symptômes notifiés. Des réunions bimensuelles se sont tenues avec les enquêteurs communautaires, les superviseurs de districts sanitaires et les leaders communautaires de chaque district pour leur présenter le nombre de cas de morsures de serpent signalés et enregistrés et les difficultés rencontrées par les enquêteurs communautaires.

La documentation des cas se faisait à l'aide de questionnaires préétablis administrés aux victimes de morsures de serpent. L'enquête visait à collecter les données socio-démographiques, les circonstances de morsure de serpent, à préciser le siège et la symptomatologie de la morsure ainsi que le type de recours aux soins. Les enquêteurs proposaient systématiquement aux victimes de se rendre à l'hôpital de district de Paoua ou de Mbaïki pour bénéficier des soins appropriés.

En cas de décès par morsure de serpent signalé aux enquêteurs, un questionnaire spécifique a été administré deux semaines après l'inhumation du corps auprès d'un proche parent de la victime défunte, décrivant les circonstances de décès et son parcours thérapeutique.

Toutes les données sur les patients ont été rendues anonymes avant leur saisie à l'aide du logiciel Microsoft Excel et analysées par Epi Info 7.

L'enquête a bénéficié de la clairance du Comité éthique et scientifique de la Faculté des sciences de la santé de Bangui par autorisation N° 17/UB/FACSS/CSCVPER/19. La collecte des données a été faite dans le strict respect de la déclaration d'Helsinki selon laquelle aucune intervention susceptible d'altérer la dignité, l'intégrité et le droit à la vie privée des patients ne sera mise en œuvre sans le consentement des patients. Les objectifs du travail étaient expliqués aux enquêtés afin d'obtenir leur consentement éclairé.

## Résultats

Un total de 412 cas de morsures de serpent a été recensé pendant la période de l’étude. En zone forestière, 198 cas de morsures de serpent ont été notifiés et 10 décès enregistrés. En zone de savane, 214 cas de morsures de serpent ont été rapportés et 2 décès enregistrés (Tableaux I et II).

**Tableau I:** Taux d'incidence de morsure de serpent et de létalité par classe d’âge Snakebite incidence rates and case fatality rates by age group

	Zone forestière				Zone de savane			
Classe d’âge	Effectif	Nombre de cas de morsure	Taux d'incidence / 100 000 hbts	Décès (%)	Effectif	Nombre de cas de morsure	Taux d'incidence/100 000 hbts	Décès (%)
0 - 14 ans	63 690	16	25	1 (6,2)	73 159	72	98	1 (1,4)
15 - 44 ans	72 581	145	200	6 (4,1)	77 695	130	167	0 (0)
45 - 59 ans	17 202	32	186	3 (9,4)	17 894	10	56	1 (10)
60 ans et plus	8 138	5	61	0 (0)	8 613	2	23	0 (0)

**Tableau II:** Répartition des cas de morsure de serpent selon les caractéristiques socio-démographiques et les types de recours aux soins Distribution of snakebite cases by socio-demographic characteristics and types of care

Caractéristiques	Zone forestière		Zone de savane	
	(n = 198)	%	(n = 214)	%
Sexe				
masculin	98	49	127	59
féminin	100	51	87	41
Activité lors de la morsure				
agricole	52	26	110	51
chasse	21	11	33	15
cueillette/ramassage	26	13	2	1
pêche	14	7	6	3
domestique	30	15	19	9
marche	44	22	29	14
collecte vin de palme	6	3	0	0
pendant le sommeil	5	3	15	7
Types de recours aux soins				
soins modernes	98	49	121	57
traitement traditionnel	100	51	93	43

La moyenne d’âge des victimes était de 26,5 ans avec un minimum de 3 ans et un maximum de 76 ans. Le sexe-ratio était de 1,5 homme pour une femme dans le DS de Paoua tandis que l’équilibre homme/femme était observé dans celui de Mbaïki.

L'incidence est nettement plus élevée chez les enfants de savane en comparaison avec la forêt et nettement plus basse à partir de

45 ans. Par contre, la létalité des enfants et des adultes jusqu’à 44 ans apparaît nettement plus élevée en zone de forêt.

Les morsures de serpent surviennent plus fréquemment lors des activités agricoles dans la zone de savane que dans la zone de forêt (26% vs 51%, p < 0,0001; Tableau II). Plus de la moitié de cas a eu recours aux soins traditionnels dans la zone de forêt tandis qu'en zone de savane, 57% des cas utilisaient les soins modernes.

Plus de 60% des cas de morsure touchent les pieds dans les deux districts sanitaires. Toutefois, la main et les doigts sont plus touchés dans la zone de savane que dans la zone de forêt (24% vs 13 %; p = 0,008; Tableau III).

**Tableau III:** Répartition des cas de morsure de serpent selon le siège de morsure et la symptomatologie clinique Distribution of snakebite cases by bite site and clinical symptomatology

Caractéristiques cliniques	Zone forestière		Zone de savane	
	(n =198)	%	(n = 214)	%
Siège de morsure				
main-doigt	26	13	51	24
avant-bras	11	6	5	2
jambe	32	16	17	8
pieds	127	64	138	65
tête	2	1	3	1
Symptomatologie				
céphalées	0	0	3	1
douleur au site de morsure	2	1	9	4
vertige	1	1	11	5
vomissement	2	1	4	2
troubles respiratoires	3	1	1	1
œdème localisé du membre	107	54	90	42
saignement au point de morsure	70	35	74	35
traces de 2 crochets	13	7	22	10

La symptomatologie est dominée par l’œdème du membre mordu, particulièrement en forêt (54% vs 42 %; p = 0,02; Tableau III).

L'absorption de décoctions était le type de recours le plus utilisé dans les deux DS (> 50%).

L'efficacité des soins traditionnels était beaucoup plus évoquée comme raison du choix de recours aux traitements traditionnels dans la zone forestière que dans la zone de savane, avec une différence statistiquement significative (p = 0,0022). En revanche, le manque de moyens financiers est la principale raison en zone de savane (p = 0,0490).

## Discussion

Notre méthodologie d'enquête par interview des parents de victimes de morsures de serpent après son signalement aux enquêteurs communautaires, présente quelques limites, car elle n'a pas encore été formellement validée par une étude comparative avec les méthodes classiques éprouvées (rétrospective à partir des registres des centres de santé, prospective ou par enquête auprès des ménages).

La méthodologie initiale prévoyait de réaliser l’étude simultanément dans les deux zones d’étude durant une période de douze mois. Des contraintes d'ordre logistique au niveau de l'organisation de l’étude et la survenue de la crise sanitaire due à la COVID-19 au courant du deuxième trimestre de l'année 2020 ont entravé le respect de cette méthodologie.

D’éventuels cas ont pu échapper aux enquêteurs lorsque ceux-ci sont restés dans les campements saisonniers agricoles, de pêche ou de chasse. Cependant, en comparaison avec la méthodologie habituelle de recueil des cas dans les registres des structures sanitaires, il est probable qu'il en ait été notifié davantage. En revanche, par rapport à une enquête active auprès des ménages, il est vraisemblable que certains cas n'aient pas été portés à notre connaissance. Contrairement à cette dernière méthode, il n'y a pas de biais de mémoire puisque tous les cas signalés ont été enquêtés pendant la période d’étude. Notre estimation du taux d'incidence se situe donc entre ces deux méthodes classiques. En outre, le questionnaire administré permet, comme pour une enquête auprès des ménages, d'obtenir davantage d'informations, notamment sur le parcours thérapeutique. L'autre avantage est l'implication de la population dans la surveillance de cette maladie tropicale négligée.

De plus, notre étude a couvert les périodes de préparation des champs et de mise en culture où le pic de l'incidence de morsures de serpent est généralement observé en zone rurale [3, 10, 19]. Guyavarch et Chippaux ont également souligné que l'enquête auprès des ménages apporte, avec une méthode simple et un protocole léger, une bonne information sur le risque de morsure ou de décès consécutif à une morsure de serpent, ainsi que sur le parcours thérapeutique [17].

Le taux d'incidence des morsures de serpent est plus élevé dans la communauté rurale de la zone de savane (respectivement 167 et 98 pour 100 000 habitants dans les groupes d’âge de 15-44 ans et 0-14 ans). Cela peut s'expliquer par le fait que cette zone de savane est réputée abriter *Echis romani* (anciennement *E. ocellatus),* une vipère à courte queue, plus agressive, décrite comme l'espèce responsable du plus grand nombre d'accidents d'envenimation et de décès à Paoua et dans toute l'Afrique subsaharienne [7, 12, 19, 33, 34]. La classe d’âge de 15-44 ans est constituée des adultes actifs dans cette zone où l'agriculture et l’élevage occupent plus de 95% de cette population, les exposant aux morsures de serpent [27]. Les enfants et adolescents de Paoua labourent les champs et chassent les petits rongeurs dans leurs trous de refuge en y mettant la main, trous qui sont également des refuges de serpents. Certains auteurs ont rapporté que les jeunes enfants sont beaucoup moins prudents lors des activités champêtres et de chasse et sont beaucoup plus victimes de morsures de serpent [16, 20, 24, 26]. Cette augmentation de l'incidence de morsure de serpent dans ces groupes d’âge a été également observée dans la région de savane du nord du Cameroun [10]. Le taux d'incidence est moins élevé en zone forestière chez les 0-14 ans et plus de 60 ans (respectivement 25 et 61 cas pour 100 000 habitants). Le taux net de fréquentation scolaire dans le DS de Mbaïki est de 4,2 contre 0,4 dans le DS de Paoua [18]. Ces enfants passent plus de temps à l’école que dans les champs et sont donc moins exposés. Par ailleurs, les plus de 60 ans sont moins actifs, et donc moins en contact avec les serpents.

Nous avons retrouvé un taux de létalité dans le DS de Mbaïki supérieur à celui du DS de Paoua (5% vs 1%, p = 0,02), ce qui est l'inverse de la situation habituelle où la létalité est plus élevée dans la zone de savane que de forêt en Afrique subsaharienne [6]. Ce fait peut s'expliquer d'une part par la présence de biais méthodologique où notre étude n'a pas couvert tous les mois du calendrier agricole, et d'autre part par la distance qui sépare les villages de l'hôpital de district dont le rayon moyen est de près de 10 km, avec l'inexistence des moyens roulants de référence [21]. Cependant, la faible létalité dans le DS de Paoua peut aussi s'expliquer par une bonne organisation de prise en charge des morsures par l’équipe de MSF au niveau de l'hôpital avec un mécanisme de référence des victimes.

Il apparaît dans notre étude que plus de la moitié des victimes de morsure de serpent dans la zone forestière avaient eu recours aux soins traditionnels avec consommation de décoctions (Tableau IV). La principale raison évoquée dans cette zone était l'efficacité de soins traditionnels, tandis que le manque de moyens financiers en est la principale raison dans la zone de savane (Tableau V). Cela peut s'expliquer par le fait que dans tout le DS de Mbaïki, il n'y avait aucune formation sanitaire périphérique disposant de la sérothérapie antivenimeuse pour la prise en charge de morsure de serpent. Ce qui a pu inciter les populations à utiliser les soins traditionnels à base des plantes médicinales, d'accès facile et de faible coût, comme l'ont également mentionné Ngbolua et collaborateurs [25].

**Tableau IV:** Types de recours aux soins traditionnels Types of traditional care

Types de soins traditionnels	Zone forestière		Zone de savane	
	(n = 100)	%	(n = 93)	%
Application de pierre noire	11	11	17	18
Application de poudre d’écorce d'arbre	12	12	20	21
Consommation de décoctions (feuilles + racines + écorce d'arbre)	57	57	46	50
Scarification	18	18	3	3
Autres (inhalation de l'urine et de l'essence)	2	2	7	8

**Tableau V:** Les raisons de choix des soins traditionnels parmi les patients y ayant recouru Reasons for choosing traditional care among patients who used it

Raisons du recours aux soins traditionnels	Zone forestière		Zone de savane		P
	(n = 100)	%	(n = 93)	%	
Manque de moyens financiers	11	11	21	23	0,049
Inefficacité des soins de médecine moderne	2	2	2	2	
Mauvais accueil dans les FOSA	2	2	2	2	
Conseils des parents et sages du village	2	2	2	2	
Distance à parcourir pour atteindre une FOSA	16	16	11	12	0,5305
Non-disponibilité des sérums antivenimeux dans les FOSA	13	13	19	20	0,2328
Efficacité des soins traditionnels	42	42	19	20	0,0022
Manque d'information	5	5	13	14	0,058
Ne sait pas	2	2	2	2	
Autre	5	5	2	2	

FOSA: Formation sanitaire

Il a été établi en Afrique que certaines plantes médicinales ont des propriétés antalgiques, anti-inflammatoires et anti-nécrotiques dans la prise en charge des morsures de serpent [11]. Il est donc souhaitable que des études phytochimiques, pharmaco-biologiques, toxicologiques et cliniques soient réalisées sur ces plantes afin d'apporter les preuves scientifiques quant à l'efficacité thérapeutique qui leur est attribuée, d'identifier de nouvelles cibles pharmacologiques et des composés bioactifs, de façon à constituer une phytochimiothèque antivenimeuse d'utilité publique, ce qui pourrait être une piste de solution alternative au coût élevé de la sérothérapie antivenimeuse dans le contexte des pays à ressources limitées. Toutefois, il convient de rappeler que 20 à 30% des morsures de serpent sont des morsures sèches ou morsures sans envenimation qui peuvent faire croire à tort à l'efficacité d'un soin traditionnel sur la guérison obtenue chez une victime [30]. En général, le parcours thérapeutique des victimes commence sur le lieu de la morsure par des soins traditionnels avant d'arriver ou non dans une formation sanitaire.

Les pieds étaient le siège de morsure le plus affecté. Toutefois, nous avons constaté que près d'un quart des morsures de serpent touche la main et les doigts dans le DS de Paoua. Cela peut s'expliquer par les activités de recherche de moyens de subsistance telles que le ramassage de noix de karité et la chasse des rats palmistes [14]. Chippaux a également décrit que le ramassage de bois, la chasse, les déplacements en particulier dans la nuit et les travaux agricoles sont responsables de 85% des morsures de serpent, circonstances qui expliquent le siège de la morsure [7].

## Conclusion

Cette étude a montré que les morsures de serpent, maladie tropicale négligée, restent un problème de santé publique en République centrafricaine de par leur incidence dans la zone de savane ainsi que dans la zone de forêt, bien que la durée de l'enquête n'ait pas couvert toutes les saisons. Cependant, la plus forte létalité dans la zone de forêt justifierait une étude des espèces et de la toxicité de leurs venins. Un cas d'envenimation par *Atractaspis irregularis* qui a un venin cardiotoxique a été décrit dans la forêt de Ngotto voisine du DS de Mbaïki [1]. Par ailleurs, il est urgent de rendre disponible les sérums antivenimeux dans les formations sanitaires périphériques de ces zones de forêt et de savane, et de développer un programme de communication sociale sur les envenimations auprès de ces populations rurales.

## Remerciements

Cette étude a été réalisée grâce à une bourse offerte par l'Organisation de coordination pour la lutte contre les endémies en Afrique centrale (OCEAC), sur la base d'une coopération financière entre la Communauté économique et monétaire de l'Afrique centrale (CEMAC) et le Ministère de la coopération économique et du développement (BMZ) de la République fédérale d'Allemagne, à travers la KfW, Banque allemande de développement.

Nous tenons également à remercier tous les enquêteurs communautaires des deux districts sanitaires de Paoua et de Mbaïki pour leur investissement dans la collecte des données de terrain de cette étude.

## Liens D'intérêts

Les auteurs ne déclarent aucun lien d'intérêt.

## Contribution Des Auteurs

R. ZARAMBAUD: conception de l’étude, recueil des données, rédaction et correction du manuscrit.

G. PIAMALE: analyse des données, relecture et correction du manuscrit.

H. S.-C. DIEMER: analyse des données, relecture et correction du manuscrit.

J. d. D. LONGO: correction du protocole de l’étude, relecture et correction du manuscrit.

G. GRESENGUET: supervision, coordination, validation du protocole d’étude, relecture, correction et validation du manuscrit.
